# “Liver–gut” axis: A target of traditional Chinese medicine for the treatment of non-alcoholic fatty liver disease

**DOI:** 10.3389/fendo.2022.1050709

**Published:** 2022-11-30

**Authors:** Kangxiao Guo, Sisheng Xu, Zhaofeng Zeng

**Affiliations:** Changsha Health Vocational College, Changsha, Hunan, China

**Keywords:** “liver–gut” axis, intestinal microorganisms, traditional Chinese medicine, NAFLD, target

## Abstract

Non-alcoholic fatty liver disease (NAFLD) occurs when fat accumulates in the liver even without excessive alcohol intake. Among the current therapeutic approaches for NAFLD, lifestyle modification with dietary changes and regular exercise is the mainstay treatment. With the rise of intestinal microecology, regulation of the “liver–gut” axis can be an effective treatment for NAFLD. This review aimed to assess the modulation of the liver–gut microbiota axis with traditional Chinese medicine (TCM) as a therapeutic approach to NAFLD and further explored its application in the newly discovered therapeutic avenues beyond NAFLD treatment.

## Introduction

Non-alcoholic fatty liver disease (NAFLD) is a clinicopathological syndrome with liver histological changes similar to alcoholic liver disease, but without a history of excessive alcohol consumption, including non-alcoholic steatosis (non-alcoholic fatty liver, NAFL) without inflammation and non-alcoholic steatohepatitis (NASH), which is associated with hepatocyte death, inflammation, and fibrosis. NAFL causes NASH, which can further evolve into liver cirrhosis; however, NASH can also sometimes lead to other outcomes. Thirty percent of patients with NASH will develop liver cirrhosis within 5–10 years. Clinical studies have shown that NAFLD is related to diabetes, hypertension, insulin resistance, abnormal liver lipid metabolism, and other factors (1). At present, NAFLD is a global epidemic trend and is one of the reasons for the increase of liver cirrhosis and liver cancer, becoming the world’s largest chronic liver disease. In Western countries, especially the United States, NAFLD has become the second leading cause of liver transplantation. However, NAFLD is not endemic to Western countries. With the increasing industrialization in Asian countries, as well as changes in lifestyle and diet, the prevalence of NAFLD in Asia has continued to increase. Li et al. (2) found that the overall prevalence of NAFLD in Asia was 29.62%. Japan had the lowest prevalence (22.28%), while Indonesia had the highest prevalence (51.04%); the general population of Mainland China had a prevalence of 29.81%. It is worth noting that the prevalence of NAFLD in Asia has been increasing year by year (25.28% in 1999–2005, 28.46% in 2006–2011, and 33.90% in 2012–2017). However, in Asia, there was no significant difference in the prevalence of NAFLD in countries with different income levels (high, upper-middle, and low), and there was no difference in the prevalence of NAFLD between rural and urban populations. Therefore, attention should also be paid to the harm caused by NAFLD in non-affluent areas, the low- and middle-income groups in Asia. In addition to its high prevalence, the harm caused by NAFLD is manifested in the fact that it can lead to liver lesions, including cirrhosis and liver cancer. At present, it is believed that NAFLD may have become another important cause of primary liver cancer after hepatitis B (HBV) and hepatitis C (HCV) virus. The annual incidence of primary liver cancer in the Asian population with NAFLD is 1.8‰, and the all-cause mortality rate is 5.3‰. The all-cause mortality of NAFLD patients can be as high as 7.3‰ (2).

Among the current treatment approaches for NAFLD, lifestyle modification with dietary changes and regular exercise is the mainstay (3), but the treatment effect is often not good and more adverse reactions are reported (4). Although promising therapeutic options targeting mechanisms for patients with NAFLD are available (5), particularly for NASH, none has been approved by the international medical community. Presently, a lot of studies have shown that Chinese medicine, relative to Western medicine, has multi-level and multi-target features, integrity, complex chemical composition, fewer adverse reactions, and a long history; therefore, a lot of scholars believe that the treatment of NAFLD using TCM has certain advantages, as long as clinical syndrome differentiation is correct, which often achieves good effects (6).

The human gut microbiota is a complex ecosystem that comprises a wide variety of bacteria, which total to approximately 1–2 kg in mass (7, 8). The gut microbiota maintains a close relationship with the host, with important roles in vitamin production, the mucosal immune system, and bacterial translocation (9). However, an overall understanding of the gut microbiota, including differences in their composition according to geographical regions, gender, and age, is yet to be established (10, 11). Theoretically, the modulation of the gut microbiota through the administration of antibiotics, probiotics, prebiotics, and symbiotics or through fecal microbiota transplantation (FMT) can be an effective approach to the treatment of NAFLD. Recent studies have released results that support this theory (12, 13).

This review aimed to assess the modulation of the liver–gut microbiota axis with traditional Chinese medicine (TCM) as a therapeutic approach for NAFLD and further explored its application in the newly discovered therapeutic avenues beyond NAFLD treatment.

## The pathogenesis of NAFLD

NAFLD is a clinicopathological syndrome characterized by fatty degeneration of liver parenchymal cells caused by alcohol consumption and other liver damage factors (e.g., drug damage, viral infection, and autoimmunity, among others) (14). Comprehensive diagnosis is mainly made through laboratory and ultrasonic examinations. The specific pathogenesis of NAFLD is presently still unclear. The “second hit” theory originated over the past decade has been widely recognized. The “first hit” is hepatic steatosis caused by insulin resistance (IR). IR refers to the decrease in the sensitivity of insulin-acting target organs to insulin; that is, a normal dose of insulin produces a fraction of the normal biological effect. This state leads to the accumulation of triacylglycerols in the liver and the decline of liver tolerance to internal and external injury factors, such as ischemia and hypoxia. In the second hit, under oxidative stress, inflammatory factors, and endotoxins, among other factors, the liver tissue shows inflammation, fibrosis, and other pathological changes, causing NASH (15). Evidence from the latest medical research has shown that the occurrence of NAFLD may also be related to poor dietary habits. A high-fat diet or large amounts of dietary fructose in the diet can cause IR and hyperleptinemia, which are accompanied by NAFLD (16). Inter-microecological imbalance is also one of the important causes of NAFLD (17). Imbalance in the intestinal flora causes damage to the intestinal mucosal barrier, increases the intestinal mucosal permeability, and induces overgrowth of some intestinal flora, including lipopolysaccharides (LPS), short-chain fatty acids (SCFAs), acetic acid, inflammatory inducers, and other harmful substances, through the intestinal barrier into the blood circulation and to the liver, thus activating the host immune system of cytokines and inflammatory mediators, forming an inflammatory response and affecting the liver (18).

## Effect of the “liver–gut” axis on NAFLD

### “Liver–gut” axis

In 1998, Marshall proposed the concept of the “liver–gut“ axis. Since then, the relationship between the gut and the liver has been receiving more attention. Studies have confirmed that the liver–gut axis is an important part of the pathogenesis of NAFLD (5). Their common embryonic origin makes the liver and the intestine closely related in terms of anatomy and biological function, and they communicate through the bile duct, portal vein, systemic circulation, and tight bidirectional connection. When the permeability of the intestinal mucosa increases, the barrier function is impaired. A lot of bacteria and endotoxins in the intestinal tract enter the portal vein system. Kupffer cells and hepatic stellate cells in the liver are activated by LPS, the main lipid components of the cell wall of Gram-negative bacteria, and the released inflammatory factors participate in the process of liver disease. These inflammatory factors can cause continuous damage to the liver, such as tumor necrosis factor alpha (TNF-α). Enterohepatic circulation is the communication system between the liver and the gut. Bile acids in the gut are recovered by the liver, secreted into the bile ducts, and then reabsorbed in the gut. Therefore, the balance of bile acids in the body determines the homeostasis of the liver–gut axis (19, 20). As important components in the gut, bile acids can inhibit the overgrowth of intestinal bacteria and affect the composition and quantity of the intestinal flora (21, 22).

### “Liver–gut” axis in non-alcoholic fatty liver disease

**Figure 1:**
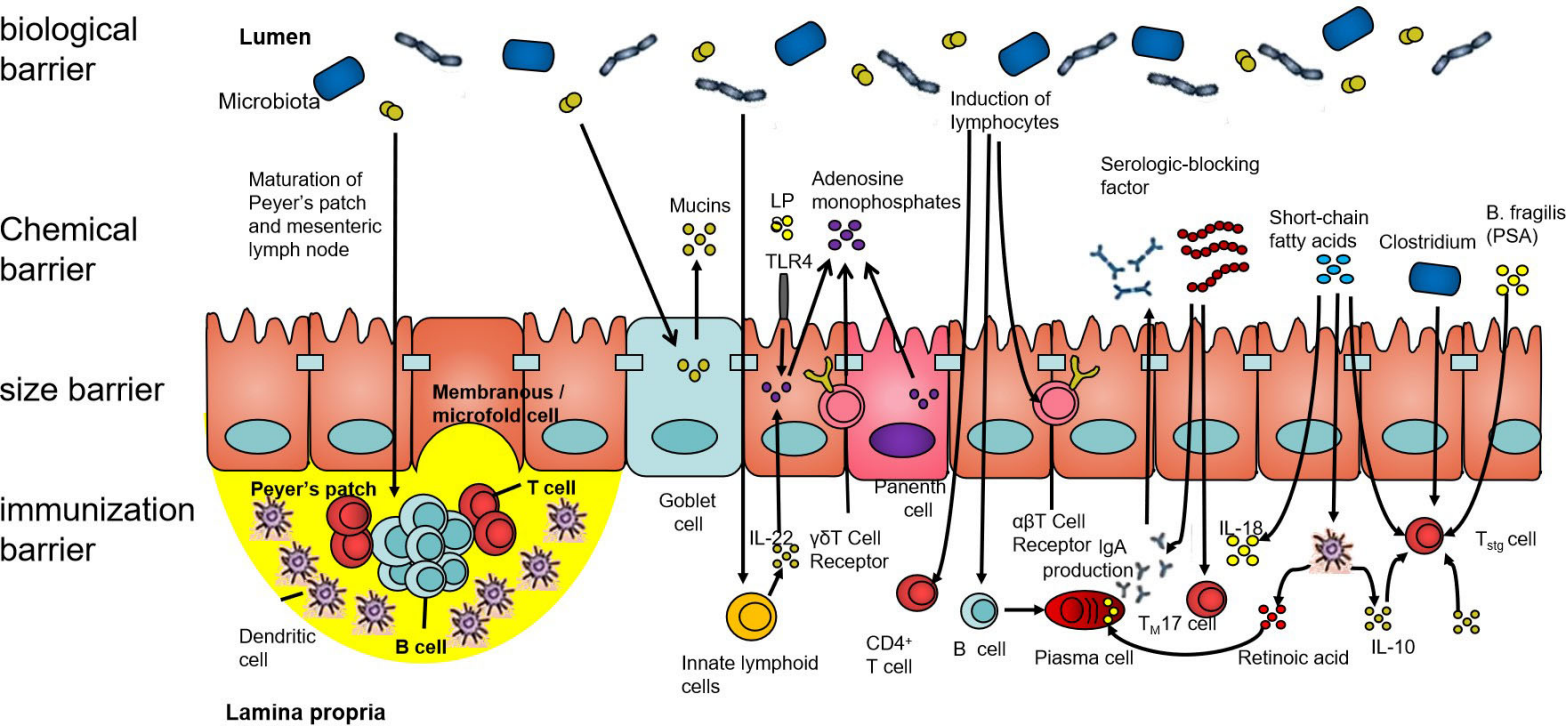
The intestinal barrier. The human intestinal tract is an important organ for the digestion and absorption of the nutrients in food. It is also a congenital barrier that maintains the balance of the intestinal environment and effectively prevents pathogenic microorganisms, their metabolites, and sensitizing substances. The intestinal barrier is composed of the biological barrier, chemical barrier, size barrier, and immunization barrier. All four barriers have different characteristics, among which, the structure of the intestinal flora forms an interdependent microbial system and interacts with other microorganisms. Its ecological balance forms the biological barrier of the human gut.

The intestinal barrier mainly comprises an immune barrier, mechanical barrier, chemical barrier, and biological barrier (as shown in **Figure 1**). The biological barrier is mainly composed of intestinal microorganisms, and these intestinal microorganisms continuously form a complex and stable microecology with the growth and development of the human body (23). The gut microbial ecosystem includes bacteria, fungi, and viruses, among others, which, together, play a role in food digestion, nutrient absorption, immune regulation, and tumor suppression, which are of great significance to maintaining the intestinal epithelial barrier function. Under the condition of stable balance, the intestinal microecology maintains the coordinated operation of the body’s organ functions. When the composition, quantity, metabolic activity, and the distribution of intestinal microbes change under different nutritional, immune, and environmental conditions, this will trigger the body’s chronic inflammatory response and even lead to a series of diseases (24).

**Figure 2:**
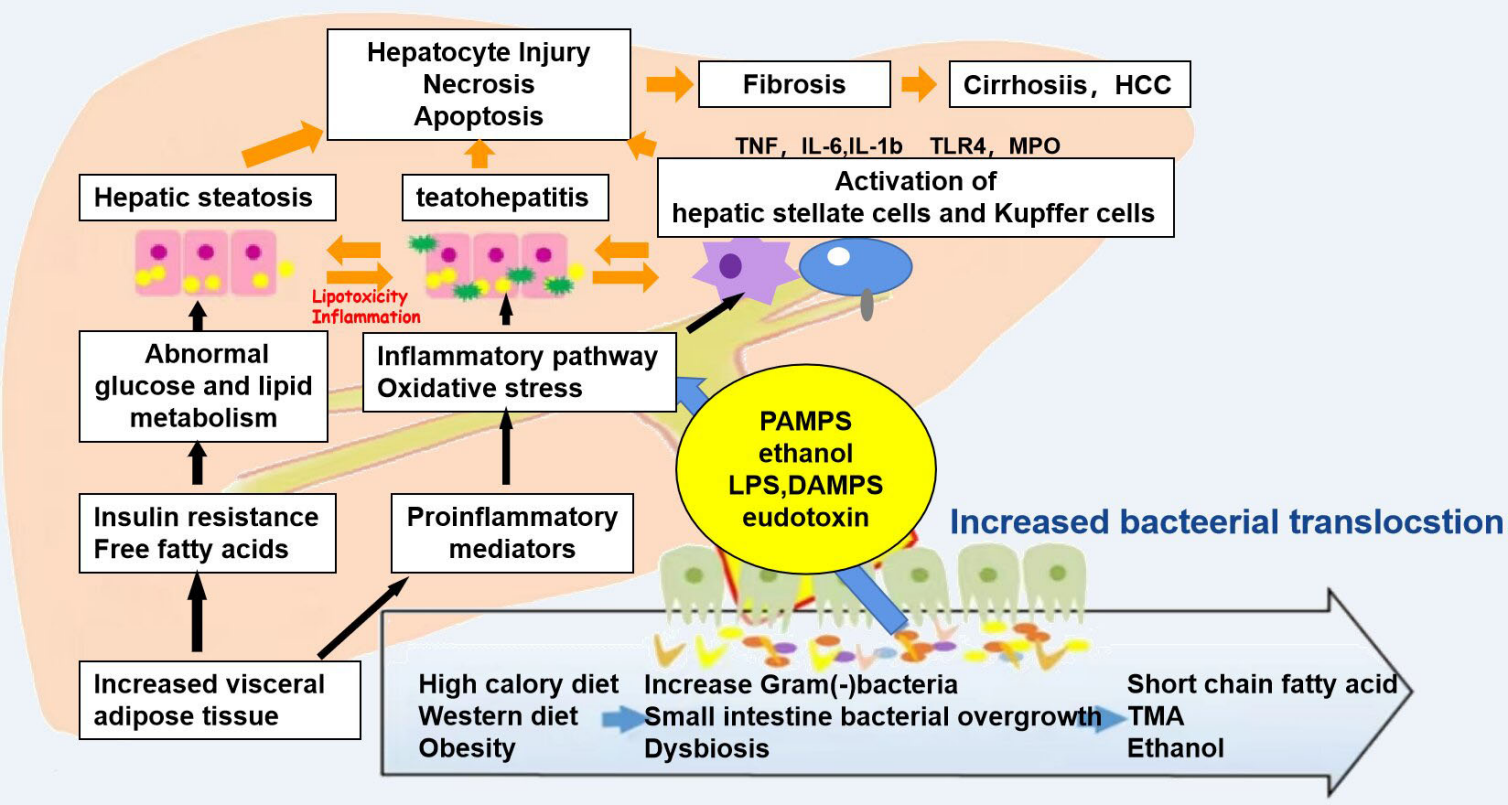
Mechanisms associated with the pathophysiology of non-alcoholic fatty liver disease (NAFLD). Diet and obesity lead to prominent changes in the microbiota, which induce intestinal bacterial overgrowth, dysbiosis, intestinal permeability, bacterial translocation, and endotoxemia, resulting in the development of NALFD.

In terms of anatomical structure and physiological function, the relationship between the liver and the intestine is inseparable. The nutrients absorbed in the intestine can be transported into the liver from the portal system through the blood, and the bile acids and other biologically active substances synthesized in the liver can also be secreted into the intestine for metabolism, thereby inhibiting the overgrowth of intestinal bacteria. When there is imbalance in the intestinal flora, the intestinal mucosal barrier is damaged and the permeability is increased. Bacteria, endotoxins, and metabolites enter the liver through the portal vein, resulting in impaired liver function; the liver then releases a series of inflammatory factors, which in turn cause intestinal damage (**Figure 2**). Yuan et al. (25) established a mouse model by strain colonization and FMT and found that *Klebsiella pneumoniae*, which produces a large amount of alcohol, can induce mitochondrial damage, impair the intestinal mucosal function, aggravate liver inflammation, and lead to the development of NAFLD. Another study compared the fecal flora of healthy groups and patients with NAFLD and observed changes in the diversity and composition of the flora, with increased proportion of Bacteroidetes and a decreased abundance of Firmicutes (26). In a study of the intestinal microbiota of 37 patients with NAFLD, Jasirwan et al. (27) found that Firmicutes, Bacteroidetes, and Proteobacteria were the predominant phyla. *Bacteroides* was more dominant than *Prevotella*, contrary to the results of previous studies on healthy populations in Indonesia. Microbiota dysbiosis was observed in most of the samples. The diversity of the gastrointestinal microbiota was significantly decreased in patients with NAFLD, high triglyceride levels, and central obesity. The Firmicutes/Bacteroidetes ratio was correlated with steatosis and obesity, whereas some of the other species in the lower taxonomy levels were mostly associated with steatosis and obesity without fibrosis. Proteobacteria was the only phylum strongly correlated with fibrosis in patients with an average body mass index. Certain gut microbes were correlated with fibrosis and steatosis. Philips et al. (28) found that the gut microbiota has a significant impact on NAFLD. Their study performed fecal transplantation in eight patients with steroid-resistant alcoholic hepatitis. It was found that the overall survival of patients who received a fecal transplant was 87.5%, while that of patients who did not receive a fecal transplant was 33.3%. One year after transplantation, bowel deformation was observed. A decrease in *Bacillus* and an increase in Firmicutes were found. Studies have shown that *Bifidobacterium* and *Lactobacillus* strains may have different effects on NAFLD and that some *Bifidobacterium* species may have protective effects in the development of NAFLD, NASH, and obesity (29). The genus *Bifidobacterium* can be considered as one of the targets for NAFLD treatment using intestinal microecology. Huang et al. fed Sprague–Dawley (SD) rats with a high-fat diet for 12 weeks to establish an NAFLD model. The study found that the liver tissue of NAFLD model rats was grayish yellow, with obvious fatty lesions, and increased cholesterol/low-density lipoprotein (LDL) cholesterol ratio and alanine (30). The activity of alanine aminotransferase (ALT) in the NAFLD group was significantly higher than that in the normal group (*p* < 0.01). The DNA of the cecal mucosa was extracted, and 16S rDNA high-throughput Illumina sequencing was used to analyze the intestinal flora of NAFLD rats. The results showed that the alpha diversity indices *S*_obs_ (observed species index), Chao, Ace and Shannon of the normal group flora were all significantly higher than those of the NAFLD model group. In the comparisons at the bacterial phylum level, the ratio of Firmicutes/Bacteroidetes in the normal group was shown to be significantly higher than that in the NAFLD model group (*p* < 0.05), while the proportion of Verrucomicrobia in the NAFLD model group was significantly increased. Comparisons at the bacterial genus level revealed that the intestinal lactobacilli in the NAFLD model group were significantly lower than those in the normal group. This study showed that NAFLD rats developed dysbacteriosis and had a decreased number of beneficial intestinal bacteria. Some studies have also found that lipid metabolism disorders can induce an imbalance in the intestinal flora, which in turn can exacerbate lipid metabolism disorders, both of which are closely related to the occurrence and development of fatty liver (31). Ley et al. (32) found that two different diets resulted in intestinal microbiological changes related to obesity and IR. IR is an important feature of NAFLD, which can be improved by antibiotic treatment (33). However, intestinal symbiotic bacteria are also very important. The study found that the severity of experimental liver fibrosis in sterile mice worsened (34). After the mice were administered small doses of antibiotics, their intestinal microbiota changed and the contents of intestinal SCFAs and cholesterol significantly increased, thus changing the fatty acid and cholesterol metabolism of the liver (35). Intestinal flora disorder can also directly affect the levels of fat factors, pro- and anti-inflammatory factors, and fat oxidation factors, thereby affecting liver metabolism and promoting liver damage (36).

## Progress in TCM treatment of NAFLD based on the regulation of the “liver–gut” axis

### Association between traditional Chinese medicine “liver and spleen theory” and “liver–gut” axis function

TCM believes that the liver belongs to wood and the spleen belongs to earth. The qi of the spleen can fill the qi and blood metaplasia and nourish the liver. Diseases of the liver transmit diseases to the spleen through the meridians, and damage in the spleen will affect the function of the liver (37). Emotional insufficiency blocks the qi movement, affecting the operation of the body’s qi, blood, and body fluids, and the liver qi stagnation accumulates for a long time. Liver stagnation and spleen deficiency are each other’s cause and effect. The transportation and transformation of the spleen depends on the normal function of the liver to disperse and relieve the spleen. Therefore, many doctors have advocated the treatment of NAFLD from the perspective of the liver and spleen, with regulation of the liver and spleen as the basic treatment method. Regulation of the liver also includes soothing it, promoting blood circulation, and promoting qi, while regulation of the spleen includes strengthening it, resolving phlegm and damp. The “Consensus on the diagnosis and treatment of non-alcoholic fatty liver diseases” pointed out that NAFLD is located in the liver and involves the spleen, stomach, and other organs. The main clinical symptoms of NAFLD include liver depression and spleen deficiency, damp-turbid internal stop, damp-heat accumulation, and phlegm and blood stasis syndrome, among others. During the development of the disease, spleen deficiency is the basic pathogenesis, as well as liver stagnation and spleen deficiency, phlegm turbidity, and internal accumulation of damp-heat, finally leading to a phlegm–damp–blood stasis inter-association. NAFLD is caused by fat deposits in the liver. Liver stagnation leads to spleen deficiency, and in the etiology and the pathological products of these syndromes, phlegm and dampness are produced by the lack of water dampness. In the final analysis, it is still a dysfunction of the spleen (38). The spleen transports and transforms water dampness. Deficiency of the spleen can lead to the lack of internal water dampness, which then leads to dampness accumulation into phlegm, and water dampness and phlegm-drinking block the normal operation of the blood. If there is luck on the side, with qi and blood circulation, this accumulation will disappear. This shows the importance of strengthening the spleen in the treatment of NAFLD.

From the perspective of modern medicine, the “spleen” in TCM theory performs the “gastrointestinal” function in the narrow sense of modern anatomy. In terms of function, the spleen, the small intestine, and the large intestine are related. The spleen is responsible for promoting clearness and transporting and transforming water and moisture, while the small intestine receives water and grains from the five internal organs and is also responsible for the secretion of clear turbidity. The large intestine, on the other hand, is responsible for conducting dregs and further absorbing body fluids. Research on the internal relationship between the “spleen and stomach–turbid poison” and the “intestinal bacteria–metabolic syndrome” has put forward the theory that, although the intestinal microecology is anatomically located in the intestine, its function belongs to the spleen (39). The liver–gut axis is proposed in modern medicine. It is believed that the intestinal barrier function is damaged, the intestinal bacteria are translocated, endotoxins enter the portal system, the immune mechanism in the liver is activated, and a large number of inflammatory factors are released, thereby causing injury in the intestinal mucosa and other organs. It can be seen through the liver–gut axis that the gut and the liver are closely related in anatomy and function, that they influence each other, and they affect the process of liver disease.

### Modern research on traditional Chinese medicine in the treatment of NAFLD based on the “liver–gut” axis

A large number of studies (12, 40, 41) have confirmed that TCM has a positive effect on the regulation of the intestinal flora, and the destruction of the intestinal microecosystem will lead to digestive disorders, poor digestion, and damage to the microecology, further leading to digestive disorders, indigestion, anorexia, and other symptoms, consistent with the liver and kidney syndrome. The use of TCMs for strengthening the spleen and dispelling dampness, such as Chinese yam, *Poria*, *Codonopsis*, and *Coix* seed, improves the spleen and stomach deficiency syndrome. Certain components of TCM can play a role in the prevention and treatment of NAFLD by improving the intestinal barrier function. Wang et al. (42) found that the *Ophiopogon japonicus* polysaccharide MDG-1 improved NAFLD in mice fed a high-fat diet by regulating the gut microbiota and hepatic lipid metabolism. Zhang et al. (43) showed that berberine can reduce liver inflammation and lipid deposition in NAFLD mice, which may be related to its role in regulating the intestinal flora. Qi et al. (44) also discovered that berberine hydrochloride can repair the intestinal mechanical barrier function of NAFLD rats and alleviate the steatosis of the liver caused by NAFLD. With the deepening of research on TCM compounds, many of these compounds based on the “liver and spleen theory” have been proven to be able to treat NAFLD by regulating the intestinal barrier. Fang (45) found that Dahuang Zexie Decoction reduced the level of pathogenic bacteria in the intestine of NAFLD rats, thereby reducing the production of LPS, regulating the function of the intestinal mechanical barrier, and improving the inflammation and lipid deposition of the liver. Cui et al. (46) believed that Xiaozhi Yigan Decoction may reduce the liver damage caused by the LPS/TLR4 pathway by improving the intestinal microbial barrier in NAFLD rats. Liver fibrosis is a serious stage in the progression of NAFLD. Chen et al. (47) found that Xiaoyao powder can improve liver fibrosis and restore part of the intestinal flora structure. Removing the spleen-invigorating drugs in the prescription weakens the effect of Xiaoyao powder on the spleen. For liver protection, Shenling Jianpiwei granules have the effect of strengthening the spleen and soothing the liver; moreover, it can reduce the expression of UCP-2 and Cytb in the liver of NAFLD mice, improve the inflammatory response and function of the liver, and protect the liver (48, 49).

In clinical practice, TCM and compound prescriptions are usually boiled in water, and the liquid formulation enters the human gastrointestinal tract after oral administration. Most of the effective ingredients are absorbed in the intestinal tract and react with intestinal microorganisms. The mechanisms of TCM compounds in NAFLD are summarized in **Table 1**. Yang et al. (50) believed that the intestinal flora including *Bifidobacterium*, *Eubacterium*, *Enterococcus*, and *Escherichia* are involved in the metabolism of saponins, a class of important active ingredients contained in TCMs such as ginseng and *Panax notoginseng*, and these substances, after the glycosylation of saponins, showed high bioavailability and biological activity. Luo et al. (51) found that rhubarb granules increased the abundance of *Bifidobacterium*, decreased *Enterobacterium* and *Enterococcus*, regulated the intestinal microecological balance, increased the expression of tight junction proteins and blocking proteins in the intestinal mucosa, and reduced enterogenous toxins such as indophenol sulfate. Xu et al. (52) collected cases of NAFLD with damp-heat accumulation. After treatment with modified Yinchen Wuling powder, the authors detected changes in the intestinal flora. The abundance of enterobacteria and *Staphylococcus* decreased, while *Bacteroides*, *Bifidobacterium*, and *Lactobacillus* increased. Yuan et al. (53) revealed that intervention with Guizhi Decoction improved high-fat diet guidance. The intestinal flora of apolipoprotein E (ApoE) knockout mice was unbalanced. After 4 weeks of treatment, the structure of the intestinal flora changed; *Bacteroides* and *Micrococcus verrucosus* increased, while the proportion of *Firmicum* decreased. Kang et al. (54) found that, in rats fed ginseng polysaccharide for a long time, butyric acid-producing bacteria such as chlamydia significantly increased, and the content of SCFAs generated in the colon of rats also significantly increased.

**Table 1:** Mechanisms of Chinese medicine compounds in non-alcoholic fatty liver disease (NAFLD).

Chinese medicine compound	Drug composition	Related findings
Liver fat-soluble particles	Astragalus, pueraria, cassia, rhubarb, orange shade, *Salvia miltiorrhiza*, seaweed, Ze diarrhea	Enhanced hepatic PPAR-α mRNA expression and decreased hepatic TNF-α expression
Eliminate turbidity and protect the liver	*Bupleurum*, red peony, white peony root, *Fructus aurantii*, xiong, licorice, *Salvia miltiorrhiza*, hawthorn, Ze diarrhea, peach kernel, safflower, gold, tylo, *Angelica*, green calyx plum	Reduced the levels of TNF-α, TNF-β_1_, and CYP2E1 in liver tissues
Add flavor to Ze diarrhea soup	Add flavor to Ze diarrhea soup	Inhibited the expression of the TLR4/NF-κB and MAPK pathway and MyD88, NF-κB p65, phospho-p65, p38 MAPK, and phospho-p38 proteins
Mercy mushroom fat oil	Mountain Ci mushroom, *Pinellia*, *Poria cocos*, *Bupleurum*, *Salvia miltiorrhiza*, *Coix* seed, *Scutellaria baicalensis*, Ze diarrhea, Chinese wolfberry, hawthorn	Regulated the JNK signaling pathway and downregulated caspase-8, FasL, and phospho-c-Jun mRNA and protein expressions
Small trapped chest soup cutting	Yellow tis, *Pinellia*, *Trichosanthes*, wood, turmeric	Downregulated GRP78 and caspase-12 protein expression
Tonifying kidney and reducing turbidity	Barbary wolfberry, virgin, *Polygonum multiflorum*, *Poria cocos*, Ze diarrhea, *Coix* seed, reed root, yam, yellow essence, Yin Chen, defeated sauce grass, licorice	Inhibited the JNK signaling pathway and decreased the JNK1, p-JNK, and p-IRS-1 protein expression
Taste water and water two elixir	Golden cherry, Gordon euryale, chinensis, astragalus	Increased the IRS-1 mRNA expression
Ginseng Ling spleen and stomach particles	North sand ginseng, *Poria cocos*, *Atractylodes*, Chinese yam, lentils, lotus seed, amomum of sand, tangerine peel, *Coix* seed, licorice	Regulated serum and hepatic TNF-α levels and decreased UCP_2_ and Cytb expression
Attached son rational soup	Ginseng, *Atractylodes*, licorice, dried ginger, aconite	The expression of TNF-α and IL-6 in SREBP-1c and FASN was down regulated
Liver corning	White snake grass, potted grass, knotweed, chinensis chinensis, *Bupleurum*, ginseng, tylo, *Salvia miltiorrhiza*, yu gold, notoginseng, green wood fragrance, licorice	Reduced the MDA and increased SOD activity
Add linglingshu soup	*Poria cocos*, cassia branch, *Atractylodes*, Dangshen, pinellia, hawthorn, herb grass, safflower, chuanxiong, *Polygonum multiflorum*, licorice	Upregulated the expression of AdipoR2 and PPAR-α
Protection of liver fat tablets	Ze diarrhea, hawthorn, lotus leaf, puhuang, notoginseng, tangerine peel	Reduced the Firmicutes/Bacteroidetes ratio and promoted the expression of claudin-1 and ZO-1 proteins
Lipid-lowering liver soup	Jersey diarrhea, cassia seed, *Salvia miltiorrhiza*, yu gold, seaweed, lotus leaves	decreased APN, TNF-α and regulated occludin and ZO- 1 protein levels

FASN, fatty acid synthase; MDA, malondialdehyde; SOD, superoxide dismutase; APN, adiponectin.

**Figure 3:**
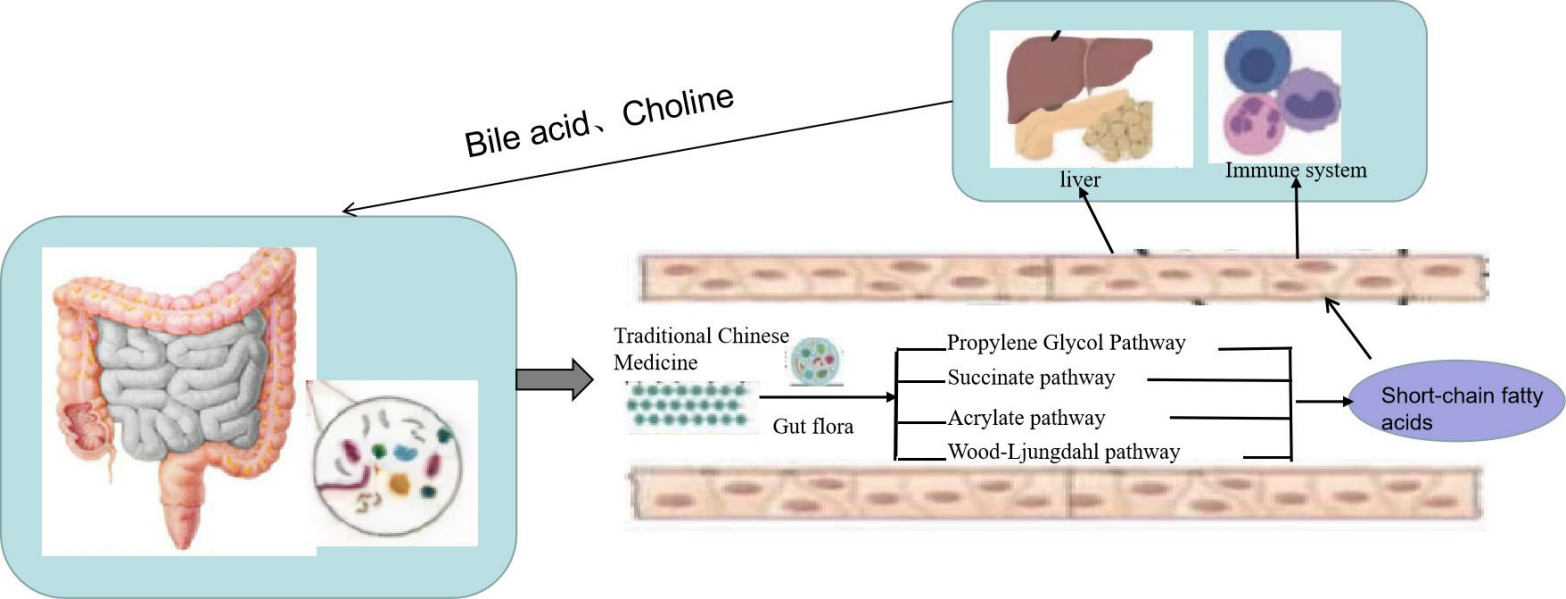
Traditional Chinese medicine (TCM) prevents non-alcoholic fatty liver disease (NAFLD) through the “liver–gut” axis. TCM enters the intestine, and intestinal microorganisms will decompose and are utilized to generate a large number of short-chain fatty acids (SCFAs). On the one hand, SCFAs can be absorbed into the blood through monocarboxylate transporters (MCTs) on intestinal epithelial cells to reach the fat, liver, and other tissues and combine with receptors on target tissues to promote fat decomposition. On the other hand, SCFAs can also stimulate intestinal endocrine cells to secrete intestinal hormone glucagon peptide 1 and casein to reach the brain, pancreas, and other tissues to inhibit appetite and energy intake.

SCFAs are a class of important metabolites produced by specific colonic anaerobes in the intestine after fermenting dietary fiber and resistant starch, mainly including acetic acid, propionic acid, and butyric acid. As important energy sources and signal molecules, SCFAs primarily transmit signals by inhibiting histone deacetylase and G-protein-coupled receptors (55). After the TCM enters the intestine, intestinal microorganisms will decompose and are utilized to generate a large number of SCFAs (**Figure 3**). On the one hand, SCFAs can be absorbed into the blood through monocarboxylate transporters (MCTs) on intestinal epithelial cells to reach the fat, liver, and other tissues and combine with receptors on target tissues to promote fat decomposition. On the other hand, SCFAs can also stimulate intestinal endocrine cells to secrete intestinal hormone glucagon peptide 1 and casein to reach the brain, pancreas, and other tissues, inhibiting appetite and energy intake (56). SCFAs can also play an anti-inflammatory role by regulating the chemotaxis of immune cells, the release of reactive oxygen species, and the number and function of intestinal regulatory T cells (Tregs) (57). Moreover, as the main energy source of intestinal epithelial cells, SCFAs can promote the proliferation and differentiation of epithelial cells, reduce cell apoptosis, promote mucin secretion, increase intestinal cross-epithelial resistance and tight connection, and reduce the intestinal mucosal permeability, thereby enhancing the intestinal epithelial barrier function, inhibiting intestinal pathogenic bacteria and intestinal endotoxin LPS from entering the body, and reducing the inflammatory response of body tissues (58). Guo et al. (59) found that the plasma LPS level was significantly negatively correlated with the content of intestinal SCFAs and that Dengzhan Shengmai could improve the intestinal barrier function by increasing SCFAs, which inhibit the entry of intestinal endotoxin LPS into the body, thereby inhibiting the activation of the liver TLR4/NF-κB inflammatory signaling pathway caused by the increase of LPS in mice fed a high-fat diet. Wang et al. (60) confirmed that *Polygonum multiflorum* could regulate the content of SCFAs in the intestine, promote the suitability of SCFAs entering the liver through portal circulation, reduce the accumulation of lipids in liver cells, organize intestinal endotoxin translocation, and effectively protect against NAFLD.

## Future perspectives

The homeostasis of the intestinal flora plays a crucial role in the maintenance of human health (61), and disturbance in the intestinal flora is closely associated with many diseases (62). At present, more and more studies have been performed on the effects of TCM on the liver–gut axis, but current research is still limited. The relationship between TCM syndromes of fatty liver and the liver–gut axis, elucidating the effect and mechanism of TCM on the intestinal flora and intestinal homeostasis, can provide a new research direction for elucidating the mechanism of TCM to provide a new research direction for clarifying the mechanism of action of traditional Chinese medicine. In addition, because the latest biotechnology can be used to detect the intestinal flora, this can improve the level of research on TCM, promote its modernization (63), and provide new ideas for the prevention and treatment of NAFLD.

## Author contributions

KG drafted the manuscript. SX researched a lot of information. ZZ critically revised the initial manuscript. All authors contributed to manuscript revision and read and approved the submitted version

## Funding

This study was supported by grants from the Natural Science Foundation of Changsha (kq2202061) and funding from Changsha Science and Technology Bureau.

## Conflict of interest

The authors declare that the research was conducted in the absence of any commercial or financial relationships that could be construed as a potential conflict of interest.

## Publisher’s note

All claims expressed in this article are solely those of the authors and do not necessarily represent those of their affiliated organizations, or those of the publisher, the editors and the reviewers. Any product that may be evaluated in this article, or claim that may be made by its manufacturer, is not guaranteed or endorsed by the publisher.

## References

[1] Luis CB Leon AA . The natural course of non-alcoholic fatty liver disease. Int J Mol Sci (2016) 17(5):774. doi: 10.3390/ijms17050774 27213358 PMC4881593

[2] Li J Zou BY Hui YY . Prevalence, incidence, and outcome of non-alcoholic fatty liver disease in Asia 1999-2019: a systematic review and meta-analysis. Lancet Gastroenterol &Hepatology (2019) 4(5):389–98. doi: 10.1016/S2468-1253(19)30039-1 30902670

[3] Sanyal AJ Chalasani N Kowdley KV McCullough A Diehl AM . Pioglitazone, vitamin e, or placebo for nonalcoholic steatohepatitis. N Engl J Med (2010) 362(18):1675–85. doi: 10.1056/NEJMc1006581 PMC2928471 20427778

[4] Guo HY Yang HR Cui GT Fang NY Deng LN Qiao F . Clinical observation of non-alcoholic fatty liver disease (liver depression and spleen deficiency syndrome). Bright traditional Chin Med (2019) 34(10):1520–2. doi: 10.13935/j.cnki.sjzx.210829

[5] Chassaing B Etienne-Mesmin L Gewirtz AT . Microbiota-liver axis in hepatic disease. Hepatology (2014) 59(1):328–39. doi: 10.1002/hep.26494 PMC4084781 23703735

[6] Wang J . Progress in TCM treatment of non-alcoholic fatty liver disease. Med Theory Pract (2017) 30(12):1747–9.

[7] Brandl K Kumar V Eckmann L . Gut-liver axis at the frontier of host-microbial interactions. Am J Physiol Gastrointest Liver Physiol (2017) 312(5):G413–9. doi: 10.1152/ajpgi.00361.2016 PMC5451561 28232456

[8] Turnbaugh PJ Hamady M Yatsunenko T Cantarel BL Duncan A Ley RE . A core gut microbiome in obese and lean twins. Nature (2009) 457(7228):480–4. doi: 10.1038/nature07540 PMC2677729 19043404

[9] Vrieze A Van Nood E Holleman F Salojärvi J Kootte RS Bartelsman JF . Transfer of intestinal microbiota from lean donors increases insulin sensitivity in individuals with metabolic syndrome. Gastroenterology (2012) 143(4):913–916 e917. doi: 10.1053/j.gastro.2012.06.031 22728514

[10] Larsen N Vogensen FK Berg FW Nielsen DS Andreasen AS Pedersen BK . Gut microbiota in human adults with type 2 diabetes differs from non-diabetic adults. PloS One (2010) 5(2):e9085. doi: 10.1371/journal.pone.0009085 20140211 PMC2816710

[11] Boursier J Diehl AM . Implication of gut microbiota in nonalcoholic fatty liver disease. PloS Pathog (2015) 11(1):e1004559. doi: 10.1152/ajpgi.00118.2019 25625278 PMC4308105

[12] He L Liu YW Guo YF Hui HY Tan ZJ . Diversity of intestinal bacterial lactases gene in antibiotics-induced diarrhea mice treated with Chinese herbs compound qi wei bai Zhu San. 3 Biotech (2018) 8(1):4. doi: 10.1007/s13205-017-1024-y PMC5718989 29242764

[13] Hui HY Wu Y Zheng T Zhou SN Tan ZJ . Bacterial characteristics in intestinal contents of antibiotic-associated diarrhea mice treated with qiweibaizhu powder. Med Sci Monit (2020) 13(26):e921771. doi: 10.12659/MSM.921771 PMC7245059 32398636

[14] Suk KT Kim MY Baik SK Gao B Gual A Lackner C . Alcoholic liver disease: treatment. World J Gastroenterol (2014) 20(36):12934–44. doi: 10.1038/s41572-018-0014-7 PMC4177474 25278689

[15] Huang ZX Yang MF Yang Z Yang YR Wang FY . Correlation between intestinal inflammation and intestinal flora imbalance in patients with nonalcoholic fatty liver disease. J Med Postgraduates (2021) 34(05):482–5. doi: 10.16571/j.cnki.1008-8199.2021.05.007

[16] Guo L Tang LQ . Progress in the pathogenesis and treatment of non-alcoholic fatty liver disease. Life Sci (2018) 30(11):1165–72. doi: 10.16286/j.1003-5052.2022.04.028

[17] Albillos A de Gottardi A Rescigno M . The gut-liver axis in liver disease: Pathophysiological basis for therapy. J Hepatol (2020) 72(3):558–77. doi: 10.1016/j.jhep 31622696

[18] Herbert T Timon EA Alexander RM . Multiple parallel hits hypothesis in nonalcoholic fatty liver Disease:Revisited after a decade. Hepatology (2021) 73(2):833–42. doi: 10.1002/hep.31518 PMC7898624 32780879

[19] Miura K Ohnishi H . Role of gut microbiota and toll-like receptors in nonalcoholic fatty liver diseas. World J Gastroenterol (2014) 20(23):7381–91. doi: 10.3748/wjg.v20.i23.7381 PMC4064083 24966608

[20] Yang YH Qin LM Zhu XP Yang XF . Mechanism of bile acid receptor mediated regulation of intestinal barrier function by bile acid. Guangdong Animal Husbandry Veterinary Sci Technol (2022) 47(04):47–54. doi: 10.19978/j.cnki.xmsy.2022.04.09

[21] Li X Peng XX Guo KX Tan ZJ . Bacterial diversity in intestinal mucosa of mice fed with dendrobium officinale and high-fat diet. 3 Biotech (2021) 11(1):22. doi: 10.1007/s13205-020-02558-x PMC7779387 33442520

[22] Guo KX Xu SS Zhang QL . Bacterial diversity in the intestinal mucosa of mice fed with asparagus extract under high-fat diet condition. 3 Biotech (2020) 10(5):228. doi: 10.1007/s13205-020-02225-1 PMC7198693 32377501

[23] He YS Tang Y Peng MJ Peng MJ Yang ZY Li WG . Influence of debaryomyces hansenii on bacterial lactase gene diversity in intestinal mucosa of mice with antibiotic-associated diarrhea. PloS One (2019) 14(12):e022580. doi: 10.1371/journal.pone.022580 PMC6897403 31809511

[24] Long CX Liu YW He L Yu R Li DD Tan ZJ . Bacterial lactase genes diversity in intestinal mucosa of dysbacterial diarrhea mice treated with qiweibaizhu powder. 3 Biotech (2018) 8(10):423. doi: 10.1007/s13205-018-1460-3 PMC6160371 30280074

[25] Yuan J Chen C Cui J Lu J Yan C Wei X . Fatty liver disease caused by high-alcohol-producing klebsiella pneumoniae. Cell Metab (2019) 30(6):1172. doi: 10.1016/j.cmet.2019.08.018 31801057

[26] Wang B Jiang X Cao M Ge JP Bao QL Tang LL . Altered fecal microbiota correlates with liver biochemistry in nonobese patients with non-alcoholic fatty liver disease. Sci Rep (2016) 6:32002. doi: 10.1038/srep32002 27550547 PMC4994089

[27] Jasirwan COM Murad A Hasan I Simadibrata M Rinaldi I . Correlation of gut Firmicutes/Bacteroidetes ratio with fibrosis and steatosis stratified by body mass index in patients with non-alcoholic fatty liver disease. Biosci Microbiota Food Health (2021) 40(1):50–8. doi: 10.12938/bmfh.2020-046 PMC7817510 33520569

[28] Philips CA Pande A Shasthry SM Jamwal KD Khillan V Chandel SS . Healthy donor fecal microbiota transplantation in steroid-ineligible severe alcoholic hepatitis: A pilot study. Clin Gastroenterol Hepatol (2017) 15(4):600–2. doi: 10.1016/j.cgh.2016.10.029 27816755

[29] Nobili V Putignani L Mosca A Chierico FD Vernocchi P Alisi A . Bifidobacteria and lactobacilli in the gut microbiome of children with non-alcoholic fatty liver disease: Which strains act as health players. Arch Med Sci (2018) 14(1):81–7. doi: 10.5114/aoms.2016.62150 PMC5778421 29379536

[30] Huang HL Zhou YJ Zheng CY Nie YQ Du JL . Changes and significance of intestinal microbiota in rats with non-alcoholic fatty liver disease [J]. Guangdong Medicine,37 (2016) 9):1283–6. doi: 10.13820/j.cnki.gdyx.20160503.005

[31] Ma CL . Effect of Lactobacillus casei on intestinal flora and lipid metabolism in high-fat diet hamsters and its mechanism. Chinese Acad Agricultural Sci (2021) 1(04):114. doi: 10.27630/d.cnki.gznky.2020.000149

[32] Ley RE Turnbaugh PJ Klein S Gordon JI . Microbial ecology:human gut microbes associated with obesity. Nature (2006) 444(7122):1022–3. doi: 10.1038/4441022a 17183309

[33] Janssen AWF Houben T Katiraei S Dijk W Boutens L Bolt N . Modulation of the gut microbiota impacts nonalcoholic fatty liver disease: a potential role for bile acids. J Lipid Res (2017) 58(7):1399–416. doi: 10.1194/jlr.M075713 PMC5496037 28533304

[34] Mazagova M Wang L Anfora AT Wissmueller M Lesley S A Miyamoto Y . Commensal microbiota is hepatoprotective and prevents liver fibrosis in mic. FASEB J (2015) 29(3):1043–55. doi: 10.1096/fj.14-259515 PMC4422368 25466902

[35] Cho I Yamanishi S Cox L Methé B A Zavadil J Li K . Antibiotics in early life alter the murine colonic microbiome and adiposity. Nature (2012) 488(7413):621–6. doi: 10.1038/nature11400 PMC3553221 22914093

[36] Torres S Fabersani E Marquez A Gauffin-Cano P . Adipose tissue inflammation and metabolic syndrome.The proactive role of probiotics. Eur J Nutr (2019) 58(1):27–43. doi: 10.1007/s00394-018-1790-2 30043184

[37] Zhang CY Liu TH Wang W . On intestinal microenvironment is an important biological basis for treating liver disease from the spleen. Chin J Traditional Chin Med (2019) 34(7):2877–80.

[38] Ma Q Shi AH Zhao Q Chen WL . Research progress in the prevention and treatment of nonalcoholic fatty liver disease by regulating mitochondrial function with traditional Chinese medicine. Chinese Journal of Traditional Chinese Medicine (2022) 47(19):5113–20. doi: 10.19540/j.cnki.cjcmm.20220704.601 36472018

[39] Wu YN Zhang L Chen T Li X Liu GX . Research progress in the relationship between human intestinal microecology and liver immunity. Chinese Journal of Microbiology (2019) 33(02):227–30. doi: 10.13381/j.cnki.cjm.202102021

[40] Li C Zhou K Xiao N Peng MJ Tan ZJ . The effect of qiweibaizhu powder crude polysaccharide on antibiotic-associated diarrhea mice is associated with restoring intestinal mucosal bacteria. Front Nutr (2022) 9:952647. doi: 10.3389/fnut.2022.952647 35873450 PMC9305308

[41] Long CX He L Guo YF Liu YW Xiao NQ Tan ZJ . Diversity of bacterial lactase genes in intestinal contents of mice with antibiotics-induced diarrhea. World J Gastroenterol (2017) 23(42):7584–93. doi: 10.3748/wjg.v23.i42.7584 PMC5698251 29204058

[42] Wang X Shi LL Wang XP Feng Y Wang Y . MDG-1, an ophiopogon polysaccharide, restrains processof non-alcoholic fatty liver disease *via* modulating the gut-liver axis. Int J Biol Macromol (2019) 141:1013–21. doi: 10.1016/j.ijbiomac.2019.09.007 31491513

[43] Yu ZH Biao YN Zhang MQ Liu CX Liu ZX Han YL . Study on the correlation between the effect of Danggui Shaoyao Powder on the prevention and treatment of nonalcoholic fatty liver and intestinal microecology. Hebei Journal of Traditional Chinese Medicine (2021) 36(04):1–5. doi: 10.16370/j.cnki.13-1214/r.2021.04.001

[44] Qi SF . Effect of berberine hydrochloride on intestinal flora in rats with nonalcoholic fatty liver disease. (Shijiazhuang: Hebei Medical University) (2017).

[45] Fang J . Research on the mechanism of treating NAFLD treatment based on the influence of intestinal flora on the mechanical barrier of intestinal mucosa. (Nanjing: Nanjing University of Traditional Chinese Medicine) (2018).

[46] Cui X . The mechanism of lipid-beneficial liver prescription prevention and treatment of high-fat diet-induced nonalcoholic fatty liver disease in rats. (Wuhan: Hubei University of Traditional Chinese Medicine) (2017).

[47] Miao J Cui HT Wang L Guo LYWang J Li P . Effects of evodiamine on carbon tetrachloride-induced liver fibrosis mice based on modulating gut microbiota. Chinese Journal of Labor Hygiene and Occupational Diseases (2021) 39(6):401–6. doi: 10.3760/CMA.J.CN121094-20201204-00666 34218553

[48] Wang CH Tao QM Wang XH Wang XR Zhang XY . Impact of high-fat diet on liver genes expression profiles in mice model of nonalcoholic fatty liver disease. Environmental Toxicology and Pharmacology (2016) 45:52–62. doi: 10.1016/j.etap.2016.05.014 27262986

[49] Yang HY Ge S . Effects of dietary fiber on obesity related intestinal microecology. Chinese food and nutrition (2020) 26(9):12–6. doi: 10.19870/j.cnki.11-3716/ts.20200713.002

[50] Yang C Liu ZD Song Y Li JB . Research progress of TCM intervention in gut microflora for prevention and control of diabetes. Chin J Exp Prescription Med (2021) 27(7):219–27. doi: 10.19763/j.cnki.2096-7403.2021.04.15

[51] Prasath LA Mohammed AZ Badreldin HA Annalisa T . The influence of the prebiotic gum acacia on the intestinal microbiome composition in rats with experimental chronic kidney disease. Biomedicine & Pharmacotherapy (2020) 133:110992. doi: 10.1016/j.biopha.2020.110992 33202283

[52] Xu SJ Xu JD Yang SZ Zhang GJ . Clinical effect of Yun Pi Hua Zhuo Granule on nonalcoholic fatty liver and its influence on intestinal flora of patients. Clinical medical research and practice (2022) 10:115–7. doi: 10.19347/j.cnki.2096-1413.202210032

[53] Yuan XW Jiang N Bai D . The regulation of immunity and intestinal flora. Chin J Exp Prescription Med (2021) 27(4):24–9. doi: 10.13422/j.cnki.syfjx.20202402

[54] Kang A Zheng X Wang GJ . Progress in the interactive regulation of active components and intestinal flora in ginseng [J]. J Nanjing Univ Traditional Chin Med (2019) 35(5):496–502. doi: 10.14148/j.issn.1672-0482.2019.0496

[55] Dalile B Van Oudenhove L Vervliet B Verbeke K . The role of short-chain fatty acids in microbiota-gut-brain communication. Nat Rev Gastroenterol Hepatol (2019) 16:461–78. doi: 10.1038/s41575-019-0157-3 31123355

[56] Canfora EE Meex RCR Venema K Blaak EE . Gut microbial metabolites in obesity, NAFLD and T2DM. Nat Rev Endocrinol (2019) 15:261–73. doi: 10.1038/s41574-019-0156-z 30670819

[57] Ohira H Tsutsui W Fujioka Y . Are short chain fatty acids in gut microbiota defensive players for inflammation and atherosclerosis? J Atheroscler Thromb (2017) 24:660–72. doi: 10.5551/jat.RV17006 PMC5517538 28552897

[58] Feng Y Wang Y Wang P Huang YL Wang FJ . Short-chain fatty acids manifest stimulative and protective effects on intestinal barrier function through the inhibition of NLRP3 inflammasome and autophagy. Cell Physiol Biochem (2018) 49:190–205. doi: 10.1159/000492853 30138914

[59] Guo HH Shen HR Zhang HJ . Dengzhan shen gmai infants nonalcoholic fatty liver disease *via* regulating endogenous microenvironment. Acta Pharm Sin (2022) 9(01):1–23. doi: 10.16438/j.0513-4870.22-0908

[60] Wang YF Lin P Lu JM Zhang M Li . Effect of radix polygoni multiflori and TSG on short-chain fatty acids in intestinal tract of NAFLD Rats. Mod Chin Med (2017) 19(9):1254–61. doi: 10.13313/j.issn.1673-4890.2017.9.009

[61] Anastasopoulos NT Lianos GD Tatsi V Karampa A Goussia A Glantzounis GK . Clinical heterogeneity in patients with non-alcoholic fatty liver disease-associated hepatocellular carcinoma. Expert Rev Gastroenterol Hepatol (2020) 14(11):1025–33. doi: 10.1080/17474124.2020.1802244 32746645

[62] Virović-Jukić L Stojsavljević-Shapeski S Forgač J Kukla M Mikolašević I. Non-alcoholic fatty liver disease - a procoagulant condition? Croat Med J (2021) 62(1):25–33. doi: 10.3325/cmj.2021.62.25 33660958 PMC7976878

[63] Wang Z Klipfell E Bennett BJ Koeth R Levison BS Dugar B . Gut flora metabolism of phosphatidylcholine promotes cardiovascular disease. Nature (2011) 472(7341):57–63. doi: 10.1038/nature09922 21475195 PMC3086762

